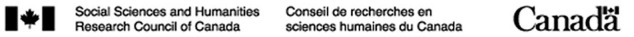# The Bangladeshi Rights and Protection of Persons With Disability Act of 2013: A Policy Analysis

**DOI:** 10.1177/10442073211066789

**Published:** 2021-12-29

**Authors:** Reshma P. Nuri, Heather M. Aldersey, Setareh Ghahari, Ahmed S. Huque, Jahan Shabnam

**Affiliations:** 1 Queen’s University, Kingston, Ontario, Canada; 2 McMaster University, Hamilton, Ontario, Canada; 3 University of Southern Denmark, Odense, Denmark

**Keywords:** children with disabilities, family needs, low-and middle-income countries, SDGs, policy analysis

## Abstract

The government of Bangladesh enacted the Rights and Protection of Persons with Disability Act of 2013 (the Act) in line with the United Nations Convention on the Rights of Persons with Disabilities. This article sheds light on the Act with particular emphasis on (a) support offered to children with disabilities (CWDs) and their families to address their needs; and (b) the extent to which the Act is in line with the international disability policy analysis framework. We compared the Act with the 18 core concepts of disability policy developed by Turnbull et al. (2001). The results affirm the government’s effort toward Sustainable Development Goals in providing support to CWDs and their families. They indicate a high degree of congruency of the Act with the core concepts. The findings also highlight the need to embrace the concepts of autonomy, confidentiality, and family-centeredness in great detail in any policy initiatives pertaining to CWDs. Furthermore, the finding shows that collaboration and coordination among ministries are imperative to achieve the goal of policies related to disability. In addition, the results highlight the need for more budgetary allocation and robust monitoring systems to track the progress of policy initiatives. As policy implementation is affected by changes in global contexts such as the coronavirus disease 2019 pandemic, policymakers in Bangladesh and other low- and middle-income countries should ensure that emergency responses are disability-inclusive and appropriate for CWDs. To ensure a disability-inclusive response, it is critical to engage individuals with disabilities and their families in meaningful consultations to identify their needs.

Bangladesh is the eighth most densely populated country in the world (1,070 population/km^2^), with an estimated population of 160.8 million (Bangladesh Bureau of Statistics, 2018). The GDP is estimated at US$274.025 billion as of 2018, with an annual growth of 7.9% (The World Bank, 2020). Over the last few decades, Bangladesh has made remarkable progress in reducing poverty. For instance, based on the international poverty line of US$1.90 a day, it reduced poverty from 44.2% in 1991 to 14.8% in 2016/17. Rapid growth enabled Bangladesh to reach lower-middle-income country status in 2015. Despite this success, the country still faces daunting challenges, as approximately 24 million people are still living below the poverty line (The World Bank, 2019).

This article focuses on policies related to children with disabilities (CWDs) in Bangladesh and aims to shed light on one important aspect of policies pertaining to persons with disabilities (PWD) more generally. Specifically, this article spotlights the Bangladeshi Rights and Protection of Persons with Disability Act of 2013, with a particular emphasis on support for CWDs and their families. It is worth noting that the Act was enacted for PWDs in general, including CWDs, and by extension, their families. For the purpose of this article, we will use PWDs when the Act addresses all PWDs (inclusive of CWDs) and use CWDs when the content of the Act is directed specifically at children. Since the purpose of this article is to assess a disability policy in relation to CWDs, the sections that follow will first provide a synopsis about the situation of CWDs in Bangladesh followed by an overview of the Rights and Protection of Persons with Disability Act of 2013, the public agency designated to oversee the implementation of the Act, and budget allocation.

## Situation of CWDs in Bangladesh

It is estimated that 57.5 million children below 18 years of age are living in Bangladesh and the estimated proportion of CWDs range from less than 1.4% (Bangladesh Bureau of Statistics, 2012) to 17.5% (UNICEF Bangladesh, 2014). These individuals are often the most marginalized and neglected groups in society. Furthermore, they may be deprived of their fundamental human rights in relation to health, education, and other resources in Bangladeshi society (UNICEF Bangladesh, 2014). For instance, only 11% of CWDs received any form of education, while the overall primary school enrollment rate in Bangladesh is 97% (UNICEF Bangladesh, n.d.), even though primary education is free and compulsory for all children according to the constitution of Bangladesh (Article 17). This is may be for several reasons, including inaccessible school premises, limited availability of disability-friendly education materials, lack of accommodation for taking exams, and shortage of skilled personnel (UNICEF Bangladesh, 2014). Such challenges forced many students with disabilities to drop out of school. Stigma surrounding disability (e.g., disability is a curse or punishment of a sin committed by parents) and discrimination at different levels of society are issues faced by CWDs (Zuurmond et al., 2015).

The government of Bangladesh signed and ratified both the United Nations Convention on the Rights of Persons with Disabilities (UNCRPD) (United Nations, 2006) and the Convention on the Rights of the Child (United Nations, 1989), thus reinforcing the country’s commitment to CWDs and bringing new attention to this population from a human rights perspective. Following these actions, the government of Bangladesh developed and enacted a number of national legislative measures (e.g., National Children Policy 2011 and Rights and Protection of Persons with Disability Act of 2013) and created initiatives to support CWDs and their families, including the provision of a disability allowance, a stipend for education and establishment of disability service and support centers in all the 64 districts of Bangladesh (Ministry of Social Welfare, 2015).

Despite all of the commitments made in international and national policies, evidence suggests that CWDs and their families experience challenges in accessing their needed support (e.g., health, education, and other resources) (UNICEF Bangladesh, 2014). Research indicates that exclusion of PWDs cannot be eliminated until and unless the government addresses exclusion in policies (Fisher & Shang, 2013; McColl et al., 2017). Specifically, McColl et al. (2017) argued that nondiscrimination legislation is critical to promote equity among citizens in terms of access to goods, services, and opportunities. More specifically, an anti-discrimination policy can help CWDs and their families to advocate for their rights and hold governments more accountable for services delivered to their citizens (Aldersey & Turnbull, 2011). This is also critical to the United Nations Sustainable Development Goal (SDG) 10, which seeks to reduce inequality by empowering and promoting social, economic and political inclusion of all (United Nations, 2015).

Therefore, this article focuses on the Bangladeshi Rights and Protection of Persons with Disability Act of 2013 to explore various support that the government of Bangladesh is offering to CWDs and their families within disability policy and also to examine how this policy is congruent with an international disability policy framework. Specifically, we sought to answer the following research questions:

**Research Question 1 (RQ1):** What support does the government of Bangladesh offer to CWDs and their families within the Rights and Protection of Persons with Disability Act of 2013?
**Research Question 2 (RQ2):** How does the Bangladeshi disability policy align with an international disability policy framework?

To answer these questions, we compared the policy document with the 18 core concepts of the U.S. disability policy as the standard for comparison (Turnbull et al., 2001). The core concepts are useful in policy analysis in that they were developed considering the needs of CWDs and their families and was also identified as a useful tool to understand family support initiatives to public policy (Turnbull et al., 2007). Furthermore, these core concepts are well aligned with the articles of the UNCRPD and have been applied by researchers to understand disability policy in other international contexts (Aldersey & Turnbull, 2011; Shogren & Turnbull, 2014). Hence, we believe that the core concepts provide a useful tool for analyzing the Bangladeshi disability policy document.

## Overview of the Rights and Protection of Persons With Disability Act of 2013

The government of Bangladesh passed the Rights and Protection of Persons with Disability Act on October 9, 2013—hereafter referred to as the “Act.” This Act repealed and replaced the hitherto Disability Welfare Act of 2001 and shifted the lens on PWDs from a welfare-based approach to a rights-based approach. The Act was enacted after significant advocacy efforts from civil society, Disabled Peoples’ Organizations and human rights activists. The status of Bangladesh as a signatory of the UNCRPD further supported the push for the enactment of the Act. The Act offers new hope regarding the situation of PWDs in that it not only establishes the rights of PWDs but also mandates the creation of different committees at the regional and national levels for better protection of their rights. Finally, the Schedule of the Act outlines specific measures that will be undertaken to protect the rights of PWDs.

## Public Agency Designated to Oversee the Implementation of the Act

The Ministry of Social Welfare is mandated as the government agency responsible for coordinating and implementing the Act. In addition, there are five committees on the Rights and Protection of Persons with Disabilities in the coordination and implementation of the Act. These committees are:

- The National Coordination Committee chaired by the Minister of Social Welfare. This committee is responsible for coordinating all disability initiatives by the government of Bangladesh. The committee also provides advice to the government relating to harmonizing national laws with the UNCRPD.
- The National Executive Committee is headed by the Secretary of the Ministry of Social Welfare. This committee is responsible for implementing the decisions adopted by the Coordination Committee.
- The District Committees, which are chaired by the Deputy Commissioners, are responsible for implementing initiatives from the National Coordination and Executive Committees. These committees are also responsible for coordinating and monitoring the activities of the Upazilla (sub-district) and Town Committees.
- The Upazilla Committees, which are directed by Upazilla Nirbahi Officers, are responsible for implementing and monitoring government disability programs throughout the Upazilla.
- The Town Committees, which are chaired by the Chief Executive Officers of City Corporations or Municipalities, are responsible to oversee government disability-related programs in their respective area.

## Budget Allocation

The content of the Act does not clearly outline the budget allocation mechanism to support the implementation of the Act. However, the national budget of the 2019–2020 fiscal year indicates that the government of Bangladesh distributes the budget for PWDs with a particular focus on the poor and the vulnerable, which is in line with the SDGs (Goal 1) (i.e., poverty reduction) (United Nations, 2015). For instance, the government allocates budget under Social Safety Net Programs for different purposes, including; (a) Allowances for the Financially Insolvent Disabled—monthly cash transfer, (b) Stipend for Disabled Students, (c) Grant for Schools for the Disabled, (d) Trust for the protection of the persons with neurodevelopmental disabilities, (e) Service and Assistance Center for Disabled, and (f) developmental projects (Ministry of Social Welfare, 2019). Evidence suggests that disability allowances made up 85.3% of the total allocation for the PWDs in the fiscal year 2019–2020 (Jahan, 2019). Furthermore, the total budget for PWDs represents 2.19% of the budget for Social Safety Nets; and 0.31% of the total budget for the fiscal year 2019–2020 (Jahan, 2019).

Taken together, the literature above suggests that the Act has potential gaps, and questions emerge regarding the suitability of the Act and its implementation. Hence, this article intends to fill these gaps by reviewing the content of the Act and answering RQ1 and RQ2 listed earlier. The article concludes with a discussion of the findings and their potential contribution to the literature.

## Method

We conducted a framework analysis of the Rights and Protection of Persons with Disability Act of 2013. The Act is written in Bengali and was independently translated into English by two authors who are proficient in both English and Bengali. The two authors then cross-checked the translations to ensure that the content and meaning of the Act were retained. Furthermore, the English translation was cross-checked with the Bengali version by another author (bilingual in English and Bengali), who randomly selected and back-translated Articles, Clauses, and Schedules in the policy document, in line with translation practices (Van Nes et al., 2010). We then imported the English version of the policy document into NVivo^TM^ 12, 2018, a computerized qualitative software package for analysis. We conducted deductive (framework) analysis (Gale et al., 2013; Ritchie & Spencer, 1994) by mapping the policy document onto the 18 core concepts of disability policy developed by Turnbull et al. (2001). We followed five steps in this deductive framework analysis; familiarization, identifying a thematic framework, indexing, charting and mapping, and interpreting (Gale et al., 2013). Two authors (R. P. N. and J. S.) who are experienced in working with CWDs and conducting qualitative research with this group independently mapped the relevant articles and clauses into the core concepts and resolved any conflict on consensus. We also kept a detailed audit trail in NVivo and shared the procedure with another author (H. M. A) to minimize bias (Pope et al., 2000).

## Results

We organized results around the two questions, which are described below, with supporting Articles and Schedules of the Act.

### RQ1: What Support Does the Government of Bangladesh Offer to CWDs and Their Families Within the Rights and Protection of Persons With Disability Act of 2013?

We found that the Act has many provisions that seek to provide individualized and appropriate services (e.g., education, health, and other services) to CWDs considering their needs and capacity. In terms of education, the Act affirms the rights of CWDs to access education through the provision of inclusive schools, providing reasonable accommodation and flexibility in the school-going age (e.g., Article 16,1.h and Schedule 9). In particular, the Act mandates access to inclusive and special education for CWDs. The Act defines inclusive education as “where students with and without disability study together” and special education refer to teaching activities that are conducted by any residential or nonresidential educational institution under special management that takes into consideration the types of disability. The Act also highlights that in a special education system, special care and services as well as preventive measures will be available. The Act, however, does not provide details on how these two educational systems should be operated.

The Act also enjoins reasonable accommodation based on the needs of students with disabilities at all levels of educational institutions (Article 16,1. N and Schedule 9. C). Furthermore, the Act recognizes the government’s responsibility in terms of offering the necessary support to CWDs in mainstream schools, including flexibility in the age of school going for CWDs (Schedule 9. A) and preparation and distribution of appropriate educational materials (Schedule 9. B). Furthermore, Schedule 9 of the Act highlights the need to contract a writer at an affordable rate for students with visual disability and/or physical disability and cerebral palsy during school examinations. It also makes provisions for fair and effective quotas on merit-based admission, a stipend to students with disabilities, and curriculum development to accommodate the needs of different types of disability. Finally, the content of the Act demonstrates a commitment to providing individualized services, wherein the government pledges to introduce special education and skill development training programs for PWDs as well as to ensure accessibility in existing technical-vocational education and training programs (Schedule 9).

In relation to health, the Act pledges PWDs’ access to the highest attainable standard of health care (Article 16. M). In particular, the Act commits the government to provide free medical services and assistive devices for PWDs who need long-term treatment and those who are also poor (Schedule 3.C). Furthermore, Schedule 3 (Clause D) mandates the provision of reduced medical expenses in private hospitals or clinics for PWDs with financial needs. Nevertheless, the Act lacks parameters to determine how such individuals qualify for free medical services and assistive devices. Finally, the Act mandates the government to take appropriate measures to prevent disability and thus emphasizes food security and nutrition for CWDs (Schedule 3. A).

Regarding other services, the Act proposes the inclusion of CWDs in the existing social safety-net and poverty alleviation program. In addition, the Act mandates reserved seats for PWDs, including children, in public transportation services. In particular, Article 32 of the Act stipulates that 5% of seats in public transport services should be reserved for PWDs. The Act also makes provision for subsidizing public transport fares to PWDs and their accompanying person (Schedule 7. D). The Act also highlights the need for the identification of CWDs. For example, the Act indicates that no child with a disability is eligible to access government support unless the child has the disability identification card issued by the government (Article 31, clause 6).

### RQ2: How Does the Bangladeshi Disability Policy Align With an International Disability Policy Framework?

We found that the Act, for the most part, reflects the core concepts of disability policy developed by Turnbull et al. (2001). In fact, 17 out of 18 core concepts are reflected in the Act. Table 1 outlines the alignment of various sections of the Act with the core concepts. The remarkably congruent core concepts that we identified in the Act were: (a) Individualized and Appropriate Services; (b) Integration; (c) Professional and System Capacity-Building; (d) Antidiscrimination; and (f) Accountability. As such, we will discuss these core concepts in detail in the section below.

**Table 1.** Comparison of the Core Concepts With the Rights and Protection of Persons With Disability Act of 2013.

Core conceptsa	Rights and Protection of Persons With Disability Act of 2013
1. Antidiscrimination	Articles 16 (Clause 1 & 2), 33 (1), 35 (Clause 1); 36 (Clause 1–11), and Schedule 15 (A–E).
2. Individualized and appropriate services	Schedules 4, 3 (D), 6, 9 (A, C, D, G, J, K), and 10 (A).
3. Classification	Articles 3, 4, 5, 6, 7, 8, 9, 10, 11, 12, 13, 14, 15, 31 (Clause1, 2, & 6), and Schedule 1.
4. Capacity-based service	Schedule 10 (F).
5. Empowerment/participatory decision-making	Schedule 16.
6. Service coordination and collaboration	Article 18 (a).
7. Protection from harm	Articles 16 (Clause 1—i & p), 29 (2); and Schedule 12.
8. Liberty	Article 16 (Clause a & l), Schedule 9 (C, D), 8(C)
9. Autonomy	None
10. Privacy and confidentiality	Article 16 (Clause1.s)
11. Integration	Article 16 (Clause 1—h, n, o, q); 34 (Clause 1 & 2); Schedule 5, 6, 9 (E) and 14
12. Productivity and contribution	Article 16 (Clause 1–i) and Schedule 10
13. Family integrity and unity	Article 16 (1.e) and Schedule 8 (E)
14. Family centeredness	Schedules 8 (A, D, & E) and 9 (I)
15. Cultural responsiveness	Definition 16, Article 16 (1.r)
16. Accountability	Articles 16 (1), 32(2), 33 (Clause 2 & 3), 35 (Clause 2), 36 (Clause 1–11), 37 (Clause 1–6), 38 (Clause 1–3), 39 (Clause 1), 40 and 41.
17. Professional and system capacity-building	Schedules 3 (E), 4 (B, C & D), 5 (A-E), 6 (A-H), 7, 8 (A, C &D) and 9 (F & I)
18. Prevention and amelioration	Schedules 3 (A &B) and 8 (B)

a Turnbull et al. (2001).

#### Individualized and appropriate services

The Act has many provisions that are aligned with the core concept of Individualized and Appropriate Services as we discussed under Question 1. These individualized services are essential to meet the needs of families of CWDs. According to Turnbull et al. (2001), these individualized services cannot be achieved unless and until the following methods are taken into consideration: (a) classification; (b) capacity-based services; (c) empowerment and participatory decision-making; and (d) service coordination and collaboration. The Act clearly classifies CWDs and their families who are eligible for these services. The Act also emphasizes an individual’s capacity in identifying and determining productive activities for PWDs (Schedule 10.F).

#### Integration

The Act places great emphasis on accessibility that is defined as “the right of persons with disabilities to get access, opportunity and treatment on an equal basis with others in all facilities and services available to the general public, including physical infrastructure, transportation, communication, information, and information and communication technology” (Article 2, Clause 13). Thus, accessibility focuses on both the built environment (e.g., disability-friendly buildings, transport, roads) and equal opportunity and treatment in receiving services. To ensure the physical accessibility of the community, the Act enjoins the inclusion of content regarding accessibility in the curriculum of Architectural programs (Schedule 5.C). To ensure equality of opportunities, the Act states that information accessibility for PWDs is necessary. This includes the availability of all publicly available information in different formats (e.g., video subtitle, audio description, screen reader, text-to-speech) based on the needs associated with different types of disabilities. Moreover, the Act affirms the rights of PWDs to receive assistive technologies and rehabilitation services (Article 16.1.O). These specialized services can facilitate PWDs to acquire the necessary skills and subsequently enable them to integrate into society. Finally, the Act mandates the provision of necessary support to PWDs to ensure their participation in sports and other recreational activities (Schedule 14).

#### Professional and system capacity-building

The Act has outlined different actions in developing system capacities and human resources. For instance, the Act mandates the establishment of new rehabilitation institutions as well as modification of existing institutions to promote institution-based rehabilitation for PWDs, especially for those who are deprived of family care (Schedule 8.C). Furthermore, the Act enjoins the development of trained personnel in the health sector: “Steps shall be taken to provide training to doctors, social workers and other health workers including the provision of medical equipment for the treatment of PWDs in government hospitals and health centers” (Schedule 3. E). Similarly, the Act highlights the provision of training to professionals and staff working in all levels of the education system (Schedule 9, F).

#### Antidiscrimination

To ensure these rights, the Act highlights steps to introduce awareness programs and campaigns to disseminate information about the capacity and contribution of the PWDs through (a) encouragement of the media to create awareness about disability; (b) incorporation of disability issues in education curricula; and (c) delivery of programs aimed at eradicating misconceptions and stereotypes about disability (Schedule 15). The Act also outlines several strategies to prohibit discrimination against PWDs (e.g., Article 33). For instance, the Act provides techniques to alleviate discrimination against PWDs in gaining admission to educational institutions. In particular, Clause 2 of Article 33 states explicitly that “the application of a person with a disability cannot be denied by the head of any educational institution based on the disability.” Article 33 further highlights the procedures in submitting complaints in a situation of discrimination against PWDs. For instance, Clause 2 of Article 33 states that “if the head of any education institution discriminates against a person with a disability during the admission process, the victim can complain to the responsible committee in this regard.” Based on the complaint, the committee will then give an order to the Chief of the institution to enroll the PWDs or can recommend the management committee to take necessary action against the Chief of the institution (Article 33).

#### Accountability

The Act outlined several strategies to ensure accountability in service provision. For instance, the Act has a provision that outlines the procedures for filing a complaint in a situation of discrimination as described earlier (see Antidiscrimination). The Act not only holds policymakers or implementers accountable to those who are affected by their actions but also has a provision to make the general population obey the law. For instance, Clause 5 of Article 37 indicates that nobody should fraudulently obtain any benefit from services that are specifically meant for PWDs.

## Discussion

The purpose of this policy analysis was two-fold: (a) understanding supports the government of Bangladesh offers to CWDs and their families in the Rights and Protection of Persons with Disability Act of 2013 and (b) assessing the extent to which the Act is aligned with the international disability policy analysis framework in addressing the needs of CWDs and their families. The results specifically highlighted that the government of Bangladesh offers a number of support to CWDs and their families in the Act (e.g., access to education, health, and social benefits). The government is also providing some tangible benefits to CWDs and families who demonstrate financial needs (e.g., disability allowance and stipend for education) (Ministry of Social Welfare, 2019). As such, the government of Bangladesh must be appreciated for its policy commitment and demonstrating some actions to make changes in the lives of CWDs and their families. Such initiatives take the government one step closer toward achieving the SDGs that manded the state’s responsibility to strengthen support to meet the specific needs of PWDs, including children (Article 23 of SDGs) (United Nations, 2015). In particular, the policy initiative to ensure equal access to the same quality of health services is aligned with the notion of leaving “no one behind” that is enshrined in the SDGs.

However, to fully support the implementation of the policy commitments, it is important to allocate an adequate budget and put mechanisms in place to monitor the progress of the Act. As disability is a cross-cutting issue, it is hard to determine the total budget that the government of Bangladesh allocates for the development of CWDs. Although the budgetary allocation for disability allowances has increased since its inception in 2006, evidence suggests that the number of beneficiaries is still below the estimated number of PWDs living in Bangladesh (World Bank Group, 2019). For instance, in the 2018–2019 fiscal budget, Bangladeshi taka (BDT) 8.4 billion (approximately US$99.57 million) was allocated for the disability allowance. This figure constitutes 1.3% of the country’s social protection budget and 0.03% of the GDP (World Bank Group, 2019). In the 2019–2020 fiscal budget, the amount increased to BDT 13.9 billion or 1,390.50 Crore (approximately US$ 164.82 million), and the number of beneficiaries is 1,545,000 (Ministry of Social Welfare, 2019). The number of recipients represents a small portion of the estimated number of PWDs—9.07% in Bangladesh (Bangladesh Bureau of Statistics, 2010).

It is important to note that CWDs and their families’ needs go beyond a disability allowance and include a need for an accessible environment that can facilitate their participation in society. In consonance with SDGs (Goal 4) (building inclusive learning environments), the Act puts great emphasis on accessible built environments in public premises. Evidence, however, suggests that public premises such as roads, schools, and public transportation are mostly inaccessible, and this can deter CWDs from participating in the mainstream society in Bangladesh (Nuri et al., 2019; Zulfiqar et al., 2018). There is not a specific budget allocation to make the built environment (e.g., roads, highways, footpaths, footbridges, and public transport) disability-friendly (Jahan, 2019). Thus, this indicates that the government’s *policy commitments* are yet to reach their intended beneficiaries *in practice*, a common problem across many low- and middle-income countries such as Tanzania (Aldersey, 2012). For instance, Aldersey (2012) found that PWDs in Tanzania have limited access to formal employment despite the government’s strong policy commitment to ensuring the rights of these individuals. Therefore, changes are required in the real world to translate the policy from aspiration to action. In particular, there is a need for specific budgetary allocation and a robust accountability mechanism to make the built environment disability-friendly. There is also a need for commitment and collaboration among relevant ministries (e.g., Ministry of Education, Ministry of Transportation, and Ministry of Social Welfares) to support the implementation of the provisions of the Act to create accessible environments in public premises.

The Act demonstrates a greater commitment to accountability mechanisms through the establishment of different committees at the national, district, and subdistrict levels and the provision of filing cases against perpetrators in a situation of discrimination. Despite all these commitments, CWDs and their families continue to experience discrimination in accessing mainstream services due to the negative attitudes of teachers and service providers (UNICEF Bangladesh, 2014). Thus, there is a need for the government to enforce nondiscriminatory policies and legislations. In particular, it is crucial to establish an independent monitoring agency with experts that can work collaboratively with other committees and monitor the progress of the implementation of the Act “on the ground” and also hold the government accountable toward the implementation of the Act. To ensure monitoring, it is critical to collect disability disaggregated data that can be used to determine whether the government is, in fact, meeting the specific requirements of the Act. The Inter-Agency and Expert Group on the SDGs proposed different indicators across all the 17 Goals that can be adopted by the monitoring agency to collect disability disaggregated data, including; the proportion of CWDs that have convenient access to public transport and social protection systems; and proportion of CWDs and families reporting situations where they felt discrimination (United Nations, 2020). Disability disaggregated data are critical to inform better public policy. For instance, a disability survey in Chile revealed that 59% of Chileans with severe disabilities have a mental or behavioral disorder. This finding informed the development of the National Mental Health Action Plan that seeks to improve the mental wellbeing of PWDs (World Health Organization, 2017). It is important to conduct a comprehensive study in Bangladesh to evaluate better if there are meaningful improvements in the situation of CWDs after the enactment of the Act.

Nevertheless, the government’s commitment to inclusive education within the Act is congruent with international frameworks such as SDGs (Goal 4), the UNCRPD, and the United Nations Educational, Scientific and Cultural Organization [UNESCO] Salamanca Statement (United Nations, 2006, 2015; UNESCO, 1994) For instance, the Salamanca Statement and Framework for Action on Special Needs Education in 1994 highlights the need for inclusive education to achieve “Education for All.” Inclusive education is critical in combating discriminatory attitudes and building an inclusive society (UNESCO, 1994). However, implementing inclusive education is challenging in many low- and middle-income countries. For instance, a recent study in Ghana revealed that teachers experience challenges such as limited availability of learning materials, limited investment from the government and a lack of knowledge and skills to address problem behaviors arising from disability to be effective teachers for student with intellectual and developmental disability (Okyere et al., 2019). Given this, there is a need for the government of Bangladesh to invest significant resources for learning materials, accessibility in school premises, including transportation, and teachers’ training. There is also a need for multisectoral collaboration among relevant Ministries (e.g., Ministry of Education and Ministry of Transportation), school boards, teachers, parents, and communities to ensure inclusion.

### Gaps in the Act and the Way Forward

When we compared and contrasted the 18 core concepts (Turnbull et al., 2001) and the Rights and Protection of Persons with Disability Act of 2013, we noted that the core concept of Autonomy is missing in the Act. We also identified that the core concepts of Privacy and Confidentiality, Family-centeredness, and Service Coordination lack detailed information. Following this, we offer possible explanations and recommendations. We also provide some strategies for incorporating these core concepts in the Act.

#### Autonomy

It is possible that this core concept may not be relevant in the Bangladeshi context because it is culturally grounded in Western perspectives (e.g., U.S. policy). Specifically, the lack of emphasis on Autonomy in the Act can be attributed to cultural reverence and trust of PWDs and their families in the expertise and skills of health care professionals in making decisions about treatment on their behalf (Talukdar et al., 2018). Furthermore, patriarchy is culturally embedded in Bangladeshi society. As a result, a male is primarily the head of the family and takes decisions about family matters, including decisions related to health care (Bedford et al., 2013). The lack of involvement of CWDs or their primary caregivers (who are mostly female) in decision-making reflects a violation of their rights to Autonomy according to international treaties (United Nations, 2006). Thus, there is the need to incorporate Autonomy in the Act as this will serve as a tool they can use to advocate for their rights. Countries like Sweden, the United Kingdom, and Australia have recently shifted their health care and social support provision systems to encourage family ownership in decision making (Shikako-Thomas & Shevell, 2018). To ensure Autonomy, it is essential to have full access to information for CWDs and their families to assist them in making informed decisions about any program that may affect them. However, in situations where the person does not have the capacity to make informed decisions, other policy procedures that were adopted in high-income countries like Canada could be useful. These include appointing a guardian to make the decision on behalf of the CWDs and their families, requesting a statutory decision-maker, or considering a qualified individual to make informed decisions regarding access to their support services (Disability Support Program Policy of 2012).

#### Privacy and confidentiality

The protection of privacy and confidentiality is critical for CWDs and their families, given that they are at high risk of exploitation of their personal information as well as dependency on multiple community-based and private agencies (Khanlou et al., 2018). As a result, the lack of protection of the privacy and confidentiality of the records of CWDs could raise the issue of violation of the rights of these individuals (Articles 22 and 31 of the CRPD). As such, the government of Bangladesh is mandated to protect the privacy of CWDs and their families, especially, those who are benefiting from their services. Khanlou et al. (2018) recommend three areas that may be useful to Bangladeshi policymakers in protecting the privacy and confidentiality of CWDs and their families. First, there is the need to raise awareness, knowledge, and skills development among various key stakeholders working with CWDs (e.g., family members, service providers) regarding the privacy rights of CWDs and their families. Second, it is essential to promote information about privacy legislation through education among key stakeholders. Finally, there is a need for government commitment to the implementation of existing legislation to allow CWDs and their families to exercise their privacy of information rights (Khanlou et al., 2018). These recommendations are supported by our analysis and are especially important in protecting the privacy and confidentiality of CWDs and their families.

#### Family-centeredness

Similarly, while family-centered support and services are important to meet the needs of CWDs and their families (Turnbull et al., 2001), the Act does not provide details of this core concept. Family-centered service is a philosophy as well as an approach to service provision to CWDs and their families (CanChild, 2003). In this approach, the family collaborates with service providers to offer culturally sensitive services and support to CWDs according to their priority needs (Rosenbaum et al., 1998). Evidence suggests that family-centered health care and particularly positive family–professional interactions can result in positive family and child-related outcomes (e.g., empowered parents and improve child’s functional skills) (Dempsey & Dunst, 2004; Dunn et al., 2012). As such, the concept of Family Centeredness should be emphasized in the Act as well as in practice. In particular, the Act should recognize the important role of family carers and incorporate the principles of helping families to balance work and care for their CWDs. There is also a need for policy action that will encourage services to address family members’ physical and mental health as these domains of health are often associated with child care (Zuurmond et al., 2015). Specifically, the Bangladeshi disability policy can be improved by incorporating issues related to respite care and also flexible work schedules for those in employment.

#### Service coordination and collaboration

As disability is a multidimensional construct, there is a need for collaboration among ministries and agencies as it is critical in providing services to CWDs and their families. Yet, the services for CWDs are often fragmented across the globe, and families may face difficulties in navigating complex systems promptly. For this reason, researchers have suggested the establishment of principles and operational rules as the key to harmonizing existing programs and resources (Shikako-Thomas & Law, 2015). Furthermore, service availability at one point can better meet the needs of families of CWDs. Thus, policymakers in Bangladesh may consider including provisions in the Act to improve service coordination. This may include designating an employee to direct or coordinate the available services for CWDs as in the case of Canada (Family Support for Children With Disabilities Act of 2003).

In sum, policy capacity is important to achieve the goals of the Act. Policy capacity focuses on the managerial and organizational abilities to inform policy decisions with evidence and its implementation with operational efficiency (Gen & Wright, 2015). Thus, there is a need for resources (e.g., budget and personnel) in formulating evidence-based policy, as it is often considered best practice in policy, and its subsequent implementation and evaluation (Forest et al., 2015; Shikako-Thomas & Law, 2015). It is also critical to engage CWDs and their families in formulating policies that are meant to serve their needs (Gen & Wright, 2015).

### Limitations

This article is not without limitations. First, we analyzed only one policy document—the Rights and Protection of Persons with Disability Act of 2013. Further analysis might include other policies related to health, education, and social support to provide a better understanding of this topic. Second, we only worked on paper-to-paper analysis without incorporating empirical research with stakeholders. Stakeholders’ perceptions about policy problems may provide a more robust and relevant picture of the reality of disability policy. In particular, the voice of policymakers is essential to get a fuller understanding of the phenomena. Their perceptions are essential to identify subsequent legislative measures and budget allocation for the PWDs after the enactment of the Act that are not readily available online. Another limitation relates to the possibility of a loss of meaning in the translation process of the Act. However, the involvement of multiple researchers who are bilingual in Bengali and English and the application of different steps ensured rigor and guarded against translation limitations. Despite these limitations, the article provides an overview of policy environments regarding how the needs of CWDs and their families are addressed in the Act and how much the Act is congruent with an international policy analysis framework. Such understanding gives an important lens toward the evolution of disability rights and policies in Bangladesh that may be relevant in other low- and middle-income country contexts.

### Conclusion

The Rights and Protection of Persons with Disability Act of 2013 reflects a commitment of the government of Bangladesh to provide support to CWDs and their families, which are in line with the SDGs (United Nations, 2015). The analysis and findings discussed in the present article make several contributions to the literature. First, it contributes to our understanding of disability policy and its implementation gaps in the Bangladeshi context specifically and low- and middle-income countries more broadly. This is because the translation of policy from aspiration to action is a common problem in many low-and middle-income countries (Aldersey, 2012). Second, the article sheds light on potential avenues for successful policy implementation targeting CWDs and their families. Specifically, the article highlights the need for more budgetary allocation and strong monitoring and accountability mechanisms to track the progress of the implementation of the policy. Third, the article outlines research gaps that need to be filled in the global literature. For instance, future studies need to explore how policy enactment influences the lives of its intended beneficiaries.

To conclude, it is imperative to reflect critically on the extent to which policy can protect its intended beneficiaries, with a long-range view, and recognize the need to take into account changes in the broader social and economic context, which may create new barriers and pressures beyond those identified in the current article. As this article is written, the coronavirus disease 2019 pandemic (COVID-19) has created a crisis globally that is also affecting Bangladesh. In the course of the pandemic, vulnerable groups, such as children, are at a higher risk of experiencing disability (Dan, 2020; Simba et al., 2020). This might arise as a result of cutting budgets from non-essential services to respond to such pandemics as well as the disruption of health services and lack of interaction with peer groups due to school closures (Simba et al., 2020; World Bank Group, 2020). As such, this can have a detrimental effect on children who already have a disability because disability-specific services such as therapy, in many cases, are considered non-essential services (Dan, 2020). Limited access to services coupled with prolonged stays in the home environment may worsen these individuals’ physical and mental health, which in turn pose additional challenges for their family members. Therefore, it is critical for policymakers to develop mitigation plans and also provide budgetary allocations to handle the challenges of the economy (e.g., GDP fall, remittance drop, and income decrease) that countries like Bangladesh will face in the actual implementation of policies targeted at CWDs, in light of long-term impacts of COVID-19 (World Bank Group, 2020).